# Social support for overcoming fear of contagion at the start of the COVID-19 pandemic. Cross-sectional study in Latin America

**DOI:** 10.3389/fpsyg.2023.1215693

**Published:** 2023-09-14

**Authors:** Irene Carrillo, Rodrigo Poblete, Piedad Serpa, Jimmy Martin-Delgado, Alejandro Giménez, José Joaquín Mira

**Affiliations:** ^1^Department of Health Psychology, Miguel Hernández University of Elche, Elche, Spain; ^2^Pontificia Universidad Católica de Chile, Facultad de Medicina, Departamento de Medicina Interna, Dirección de Calidad y Gestión Clínica Red de Salud UC CHRISTUS, Santiago, Chile; ^3^Department of Clinical Management and Patient Safety, School of Medicine, Universidad de Santander, Bucaramanga, Colombia; ^4^Hospital Luis Vernaza, Junta de Beneficencia de Guayaquil, Guayaquil, Ecuador; ^5^Instituto de Investigación e Innovación en Salud Integral, Facultad de Ciencias Médicas, Universidad Católica de Santiago de Guayaquil, Guayaquil, Ecuador; ^6^ATENEA Research Group, Foundation for the Promotion of Health and Biomedical Research of Valencia Region (FISABIO), Sant Joan d’Alacant, Spain; ^7^Hospital Provincial-Pla Health District, Health Department of Alicante-Sant Joan, Alicante, Spain

**Keywords:** healthcare professionals, COVID-19, social support, community support, acute stress

## Abstract

**Background:**

The psychological impact of the COVID-19 pandemic on healthcare professionals has been widely studied, along with different strategies to minimize it. However, professionals’ assessment of the social support received and the factors that mitigated their fear of contagion have not been described. This study aimed to assess healthcare professionals’ satisfaction with the social support and information received and their efforts to self-isolate to avoid infecting loved ones in Chile, Colombia, and Ecuador.

**Methods:**

A cross-sectional online survey, conducted from July to September 2020 in three Latin American countries, elicited healthcare professionals’ satisfaction with social support from colleagues, their community, the media, and scientific societies; as well as the information received about the evolution of the pandemic and measures to avoid contagion. The EASE scale was used to measure acute stress.

**Results:**

Survey responses were received from 700 professionals. The response rate per country exceeded the estimated sample size except in the case of Colombia, which was 81.4%. In general, peer support was highly valued, though satisfaction was lower in high-risk units (*p* < 0.001). Those who directly assisted COVID-19 patients perceived the least community support (*p* = 0.023). Professionals from high-risk units (*p* = 0.013) and those who experienced greater acute stress (*p* = 0.05) assigned the lowest rating to the information offered by the centre on the pandemic. Men perceived more support from colleagues and better information from the centre than women (*p* < 0.05). Just 10.7% of professionals changed their residence during the pandemic, but those who worked in high-risk areas self-isolated more frequently (*p* = 0.026).

**Conclusion:**

In the early stages of the COVID-19 pandemic, healthcare professionals in Chile, Colombia, and Ecuador greatly valued the support received from their peers. Being infected with COVID-19, working in high-risk areas, experiencing higher self-reported acute stress, and having an infected co-worker were predictors for self-isolation to protect their relatives. These results point to the appropriateness of putting in place institutional resources based on peer support and specific communication strategies and action protocols to build resilience and responsiveness to future health crises.

## 1. Introduction

The COVID-19 pandemic shook societies and health systems around the world. The geographic variability in the intensity and evolution of the impact of this crisis was subject to diverse conditioning factors. The Latin American and Caribbean region was one of the most affected worldwide. According to data from the Coronavirus Resource Center of the Johns Hopkins University & Medicine, infections in this region accounted for 12.3% of total cases and 25.7% of COVID-19 deaths worldwide (Johns Hopkins University & Medicine, 2023). These figures highlight the lethality of coronavirus in this region, although differences between countries were also observed across Latin America and the Caribbean. This variability may be determined, in part, by differences in health infrastructure, political leadership, poverty, and inequality in access to resources (health care, PPE, vaccines, etc.), such as that suffered by the indigenous population or underserved regions (Martin-Delgado et al., 2020; LaRotta et al., 2023). The pandemic affected, to a greater extent, those countries with pre-existing gaps in access to quality healthcare. The increase in clinical risks and the deterioration in patient safety and quality of care caused by COVID-19 was more intense in those regions with less capacity to respond to this type of eventuality. In this regard, Latin America, with Central Europe, has the highest amenable mortality rate due to the receipt of poor-quality health services (Garcia Elorrio et al., 2021).

The first case of COVID-19 in Latin America was confirmed on February 26, 2020, in Brazil (Pan American Health Organization, 2020). One day later, the first case was detected in Ecuador, a 71-year-old Ecuadorian woman who had arrived from Spain 2 weeks earlier (Gobierno de la República del Ecuador, 2020). On March 3, the first case was confirmed in Chile in a 33-year-old man returning from a trip to Southeast Asia (Ministerio de Salud Gobierno de Chile, 2020). Three days later, a 19-year-old woman from Italy tested positive for COVID-19 in Colombia (Ministerio de Salud y Protección Social, 2020).

During the first waves of the COVID-19 pandemic in 2020, healthcare professionals experienced increased compassion fatigue (Ruiz-Fernández et al., 2020), acute stress (Martin-Delgado et al., 2022), and psychosomatic and anxious-depressive symptoms (Xiong et al., 2022) due to the pressure and working conditions they were under. Recent studies show that the decline in the quality of life and well-being of healthcare professionals persists 1 year after the outbreak of the COVID-19 pandemic (Rania et al., 2023).

Zhang et al. (2022) analyzed the psychological symptoms of the Latin American population during the COVID-19 pandemic in a systematic review and meta-analysis that included 62 studies and 196,950 participants. The prevalence of anxiety (35%), depression (35%), distress (32%), and insomnia (35%) were higher in South America than in Central America and among frontline healthcare professionals and university students compared to the general population and general healthcare professionals. The greater vulnerability of frontline professionals has been associated with increased risk of infection, burnout, direct exposure to patient suffering or death that is related to compassion fatigue and secondary trauma, and fear of COVID-19 infection and of being the source of family contagion. Another meta-analysis conducted exclusively on Latin American healthcare professionals showed that being female, younger, working shifts of more than 12 h, working in the ICU or surgery and being close to the epicenter of the outbreak increased the probability of developing mental health disorders and worse mental health outcomes (Rosales Vaca et al., 2022). Between 7.2 and 43.8% of the professionals presented symptoms of post-traumatic stress disorder, 49.8% burnout and 64.3% compassion fatigue (Samaniego et al., 2020; Juárez-García et al., 2021). In this population, moderate and severe levels of depression and anxiety were related to suicidal ideation (Restrepo-Martínez et al., 2021).

Fear related to COVID-19 is one of the determinants of psychological distress resulting from the pandemic (Rosales Vaca et al., 2022). The fear of becoming infected by the virus or carrying it home was particularly frequent among healthcare professionals because of their exposure to the biological risk of being in continuous contact with patients infected by COVID-19 (Mira et al., 2020a; Labrague and de Los Santos, 2021). This element clearly differentiates this pandemic from other previously studied health emergencies, such as natural disasters, terrorist attacks, armed conflicts, and even other epidemics that have not had the same global reach as the novel SARS-CoV-2. Fear and risk perception were associated with prevention behaviors such as hand washing (Commodari et al., 2020; Bonilla-Asalde et al., 2023). Fear could initially be an adaptive coping strategy by motivating the adoption of preventive behaviors, but it eventually affects the well-being and clinical decision-making capacity of professionals (Becerra-Medina et al., 2022; Rosales Vaca et al., 2022) and could deteriorate the quality of patient care (Schiess et al., 2021).

Social support seems to play an influential role as a protective factor against the fear of contagion and psychological distress, despite the little interest it has received in the scientific literature regarding healthcare professionals during the COVID-19 pandemic. In this line, Beck and Daniels (2023) found, in a sample of 342 healthcare professionals from the UK National Health Service, that higher intolerance of uncertainty, intense fear of contagion, and lower levels of perceived social support predicted psychological distress. In some cases, healthcare professionals have suffered from the absence of social support due to the stigma associated with their profession, whereby others perceived them as vehicles of the virus (Taylor et al., 2020). This social stigma has also contributed to the deterioration of their mental health (Badrfam et al., 2022).

Studies in the general population have highlighted the value of community social support during the COVID-19 pandemic, especially during periods of lockdown policies. Jia et al. (2021) observed that the psychological well-being of women in rural settings was most affected by social isolation measures. Community productive capacity, community cohesion, and access to basic medical services, among other factors, reduced pandemic-derived risks to people’s mental health. Community support and identity were also key in the collective response to the health crisis caused by SARS-CoV-2. Thus, Stevenson et al. (2021) found that the preexistence of community identification predicted lockdown compliance and helping behavior during the crisis, increasing the perception of neighborly support. These authors concluded that the perception of support availability facilitates providing and receiving support in response to collective crises.

Everly et al. (2021) described the predictable psychological pattern of community response to disasters or critical situations. They identified the following five phases: impact (panic, confusion), heroic (altruism, high level of activity), honeymoon (community cohesion, optimism), disillusionment (community stress and fatigue, feeling of abandonment), and restoration (working through grief, adjusting, and rebuilding). Understanding these phases can help anticipate the support needs of particularly vulnerable groups, such as healthcare professionals, during critical situations, such as the COVID-19 pandemic.

Health authorities’ responses to this situation, both at the national and local levels, sought to shore up the morale and response capacity of these personnel (Petrella et al., 2021), aware that the loss of this line could truncate the response capacity to the coronavirus pandemic by society. Support through psychological first aid (Asaoka et al., 2021), 24/7 call lines (Fukuti et al., 2021), briefing techniques (Azizoddin et al., 2020), and self-rescue using apps (Mira et al., 2020b) are just a few of the most common responses (López-Pineda et al., 2022), along with solidarity campaigns from the community. Likewise, different initiatives have been launched to counteract fear of contagion, such as setting up hotel rooms or residences for professionals to spend the night temporarily away from home or offering specific instructions on what to do when returning home from work (Vimercati et al., 2020).

In Latin America, the collaborative governance index proposed by Cyr et al. (2021) suggests that governments that promoted collaborative governance have been more effective in containing the mortality rate during the pandemic. This collaboration translated into cooperating on resource management, preparing for the expected exponential growth of cases, and conveying a unified message to the population to prevent further outbreaks. The analysis of Knaul et al. (2022) demonstrates the lack of homogeneity in the adoption of public health measures at the national and subnational levels in Latin America and reinforces the need for national governments to coordinate among countries and with their subnational governments to adapt the local response to the crisis to the changing conditions of each place.

The available research evidences the major impact that the COVID-19 pandemic had in Latin America. The mortality rate highlights pre-existing weaknesses in the health systems of this region. The variability between and within countries also highlights the effect of differences in government-driven pandemic management. Many studies have reported the impact of this situation on the mental health of healthcare professionals. In this context, fear of contagion and the role of perceived social support stand out as relevant variables. However, there is little literature on professionals’ own assessments of the social support they had access to or of the means available to counteract the fear of contagion, particularly in Latin American countries (Karagöl and Törenli Kaya, 2022).

This study assessed healthcare professionals’ satisfaction with the social support and information received and their efforts to self-isolate to avoid infecting loved ones in three Latin American countries.

## 2. Materials and methods

This cross-sectional study was conducted from July to September 2020, a period which saw 919,744 diagnosed cases of COVID-19 in Colombia, 544,644 in Chile, and 213,724 in Ecuador (World Health Organization, 2020). This research project was risk-free, using an online questionnaire with voluntary and anonymous participation designed in accordance with the Declaration of Helsinki. This study was approved by the Pontific Catholic University of Chile Ethics Committee for Health Sciences (200630029) and complies with Resolution 8,430 of 1993 of Colombia (Burns et al., 2008).

### 2.1. Instruments

The research team developed a set of survey items to explore healthcare professionals’ satisfaction with the social support received from colleagues, their community, the media, and scientific societies, along with the information received on the evolution of the pandemic and measures to avoid contagion, such as alternative residential facilities. Respondents were asked to rate their satisfaction on each item on a scale of 0–10, with higher scores indicating more satisfaction. The Self-applied Acute Stress Scale (EASE) scale was used to assess acute stress levels perceived by professionals (Mira et al., 2021). The EASE is a validated scale designed to assess acute stress in healthcare workers caring for COVID-19 patients. The scale presents good internal consistency (Cronbach *α* = 0.85 and McDonald *ω* = 0.87) and is made up of 10 items that evaluate two dimensions (affective response and fear/anxiety response) with a Likert-type response scale of 4 points. The range of total scores is 0–30 points. Participants who scored 25 or more were under acute stress.

The survey was carried out using SurveyMonkey, a web-based application that allows only a single response for each internet protocol address. Up to four reminders to respond were sent to participants in each country.

### 2.2. Participants

A snowball sampling approach was used to recruit healthcare professionals from participating hospitals, clinics, and ambulatory care centers. This non-random, purposeful sample was invited from 13 July to 30 September 2020, using institutional emails and instant messaging applications. All participants were informed of the purpose of the study, the voluntary nature of participation, and the expected utility of their responses. For a precision of 2% and a confidence level of 95%, the minimum number of responses needed was 119 from professionals in Chile, 312 from Colombia, and 104 in Ecuador—a sample size proportional to the country’s population size. Representation of at least 10% was sought from all major cadres of healthcare professional groups, including general practitioners (6 years training), specialized medical doctors (4 years additional training), nurses (5 years training), auxiliary nurses (2 years training), and other professionals (psychologists, physiotherapists, and respiratory therapists).

### 2.3. Measures and data analysis

The primary measures assessed were satisfaction with the social support and information received, self-isolation patterns and availability of residential facilities, and acute stress.

A descriptive analysis of the quantitative variables was undertaken, with results expressed as means with 95% confidence intervals (CI). Categorical variables were presented as frequencies, and the chi-square statistic and Fisher’s exact test were applied, as appropriate, to compare groups by professional cadre, sex, exposure to risk of infection, and country. For the analysis of the work area variable, two categories were generated: low-risk (non-COVID hospital ward, outpatient clinic, middle managers, and others) and high-risk (specific area for COVID patients, critical care, emergencies, and home service and/or ambulance). In addition, the Mann–Whitney *U* test and the Kruskal–Wallis test were applied to compare the quantitative variables. A stepwise binary logistic regression was performed using the Wald statistic. Statistical significance was accepted at confidence intervals of *p* < 0.05 and 95%. The statistical analysis of the sample was carried out using the SPSS software version 28.0.0.0.

## 3. Results

A total of 700 professionals responded to the survey during the study period. The overall response rate more than doubled the sample size estimation. However, the survey participation analysis by country showed a lower response than the estimated sample size in the case of Colombia (response rate of 81.4%). Most of the respondents were women (*n* = 504, 72%; Table 1). By professional groups, 312 (44.6%) were doctors, 217 (31%) nurses, and 171 (23.3%) other health professions. A quarter (*n* = 175) confirmed that they had been infected. The proportion of professionals reporting infections among relatives was higher in Ecuador (97.7%) than in Chile (31.4%) or Colombia (85.7%) (*p* < 0.001). The same pattern was apparent in colleagues of infected professionals (Ecuador 95.3%, Chile 29.7%, Colombia 78.6%, *p* < 0.001).

**Table 1:** Sample description.

Variables	*N*	%
Country		
Chile	336	48.0
Colombia	254	36.3
Ecuador	110	15.7
Sex		
Male	195	27.9
Female	504	72.0
Age, mean (95% CI) [range]	36.2 (35.5–36.9)	[17–70]
Current position		
General practitioner	180	25.7
Specialist physician	132	18.9
Nurse	217	31.0
Nursing assistant	91	13.0
Others	80	10.3
Work area		
Non-COVID-19 hospital ward	70	10.0
Specific area for COVID-19 patients	83	11.9
Critical care (ICU or intermediate)	145	20.7
Outpatient clinic	208	29.7
Middle management	40	5.7
Emergency department	109	15.6
Home care and/or ambulance care	30	4.3
Other	15	2.1
Have you been infected with COVID-19 during the pandemic?		
No	390	55.7
Yes	175	25.0
I do not know	135	19.3
COVID-19 cases among family members during the pandemic		
Other than patients, I know of no cases	299	42.7
Someone I know has been infected with a mild case	229	32.7
Someone I know has been infected with a severe case	172	24.6
COVID-19 cases among colleagues during the pandemic		
Other than patients, I know of no cases	347	49.6
A colleague has been infected with a mild case	256	36.6
A colleague has been infected with a severe case	97	13.9
Acute stress		
High	16	2.3
Mild to moderate	684	97.7

### 3.1. Support among colleagues and from other social groups

Professionals highly valued the support they received from colleagues from the same service or unit to help them cope with the pandemic situation. Table 2 shows the results according to the risk category and country. Satisfaction with the support received from colleagues in other services/units was higher in male versus female hospital doctors (7.5, 95% CI 6.9–8.1 versus 6.2, 95% CI 5.7–6.8, *p* = 0.006). In this group, men valued the support received from their colleagues more than women (8.8, 95% CI 8.4–9.3 versus 7.2, 95% CI 6.5–7.8, *p* < 0.001). General practitioners in outpatient clinics valued the support they received from colleagues in other services/units (6.8, 95% CI 6.3–7.2) more positively than nurses working in hospitals (5.9 95% CI 5.5–6.3, *p* = 0.031).

**Table 2:** Professionals’ satisfaction with social support and information on the pandemic, by risk area and country (scale: 0–10, with higher scores indicating more satisfaction).

	Satisfaction with perceived support, mean (95% CI)				Satisfaction with information received on the pandemic, mean (95% CI)	
	From colleagues in my service/unit	From colleagues in other services/units	From my community (neighbors, friends)	From society at large (community, media, scientific societies)	From my hospital/center	From my service
Overall score (*N* = 700)	7.5 (7.3–7.7)	6.3 (6.1–6.5)	5.7 (5.5–6.0)	5.0 (4.8–5.2)	6.6 (6.4–6.8)	6.7 (6.5–6.9)
Risk category						
Low-risk area (*N* = 333)	7.6 (7.3–7.9)	6.7 (6.5–7.0)	6.0 (5.7–6.3)	5.2 (4.9–5.5)	6.9 (6.6–7.2)	6.9 (6.6–7.2)
High-risk area (*N* = 367)	7.5 (7.2–7.8)	5.8 (5.5–6.2)	5.5 (5.1–5.8)	4.8 (4.5–5.1)	6.3 (6.0–6.6)	6.5 (6.2–6.8)
*P*-value	0.64	<0.001	0.029	0.069	0.013	0.26
Country						
Chile (*N* = 336)	7.9 (7.6–8.1)	6.2 (5.9–6.6)	6.0 (5.7–6.4)	5.1 (4.7–5.4)	6.8 (6.5–7.1)	6.9 (6.6–7.2)
Ecuador (*N* = 110)	7.1 (6.6–7.7)	6.0 (5.5–6.6)	5.6 (5.1–6.2)	4.9 (4.3–5.5)	5.9 (5.3–6.4)	6.2 (5.7–6.7)
Colombia (*N* = 254)	7.3 (7.0–7.6)	6.4 (6.1–6.8)	5.4 (5.0–5.7)	4.9 (4.5–5.2)	6.7 (6.3–7.0)	6.6 (6.2–7.0)
*P-*value	0.003	0.56	0.020	0.69	0.007	0.022

Professionals assigned to high-risk units expressed less satisfaction with the support received from colleagues and the community than those in low-risk services (Table 2). In particular, the professionals working in critical care units assigned the lowest ratings to the support received from other services/units (5.6, 95% CI 5.1–6.2; *p* = 0.033), while professionals in the COVID-19–specific areas perceived the least support from the community (4.6 95% CI 3.9–5.3; *p* = 0.025). Professionals who reported being infected with COVID-19 had the lowest opinion of the support received from society (infected: 4.9, 95% CI 4.5–5.4; not infected: 5.2, 95% CI 4.9–5.5; unknown infection status: 4.4 95% CI 3.8–4.9; *p* = 0.023). Having a colleague who was infected was associated with lower ratings of the support received from colleagues, regardless of the severity of the disease (no colleague infected: 7.9, 95% CI 7.6–8.2, colleague with mild infection: 7.2, 95% CI 6.9–7.6; colleague with severe infection: 7.0, 95% CI 6.4–7.6 *p* = 0.004).

According to country of residence (Table 2), Chilean professionals expressed greater satisfaction with the support received from their unit colleagues (*p* = 0.003) and their community (*p* = 0.020). Among the professionals who were infected in these countries, we found no differences in satisfaction with social support or information received, nor in the level of acute stress reported.

### 3.2. Information on the evolution of the pandemic

Professionals in high-risk areas had a poorer perception of the information about the pandemic received by the hospital/center than those in low-risk services (Table 2). Overall, respondents assigned the information received with a mean score of 6.6 points.

Among hospital doctors, men were more satisfied than women with the information received in their service/unit about the evolution of the pandemic (7.4 95% CI 6.6–8.2 versus 6.3 95% CI 5.7–7.0, *p* = 0.029). Professionals in Ecuador expressed less satisfaction with the information received, both in their hospital/center (5.9, 95% CI 5.3–6.4, *p* = 0.007) and in their service (6.2, 95% CI 5.7–6.7, *p* = 0.022; Table 2). Compared to their female counterparts, male professionals in Chile reported greater satisfaction with the information provided by their hospital/center (7.5, 95% CI 7.0–8.1 versus 6.6, 95% CI 6.3–7.0, *p* = 0.021).

The experience of being infected with COVID-19 was related to worse satisfaction with the information received on the pandemic situation in the hospital/center (infected: 6.4 95% CI 6.0–6.8; not infected: 6.9 95% CI 6.6–7.2; unknown infection status: 6.1 95% CI 5.6–6.6; *p* = 0.012). Professionals with no infected colleagues had a better opinion of the information received from the service (7.1, 95% CI 6.8–7.3) compared to those who had colleague with a mild (6.4, 95% CI 6.0–6.7) or severe infection (6.3, 95% CI 5.7–6.8; *p* = 0.005). A similar pattern was observed regarding the information received from the hospital/center (no infected colleague: 6.9, 95% CI 6.6–7.2; colleague with mild infection: 6.4, 95% CI 6.1–6.8; colleague with severe infection: 6.1, 95% CI 5.5–6.7; *p* = 0.033).

### 3.3. Self-isolation measures

A total of 292 (41.7%) professionals did not consider changing their place of residence in the early phase of the pandemic (Table 3). This decision was more frequent among healthcare professionals working in low-risk areas (45.6% versus 38.1%, *p* = 0.046). It was also more frequent among professionals in Chile (45.2%) and Colombia (44.9%) compared to Ecuador (23.6%) (*p* < 0.001).

**Table 3:** Self-isolation patterns among professionals, by risk area and country (*N* = 700).

	Changed residence *n* (%)	Changed bedrooms to self-isolate from loved ones *n* (%)	Stayed in a residential facility for professionals *n* (%)	Considered but did not make residential changes *n* (%)	Did not make or consider any residential changes *n* (%)
Total (*N* = 700)	75 (10.7)	165 (23.6)	14 (2.0)	154 (22.0)	292 (41.7)
Risk category					
Low-risk area (*N* = 333)	37 (11.1)	66 (19.8)	5 (1.5)	73 (21.9)	152 (45.6)
High-risk area (*N* = 367)	38 (10.4)	99 (27.0)	9 (2.5)	81 (22.1)	140 (38.1)
*P-*value	0.81	0.026	0.43	1.00	0.046
Country	Residential facility available				
Chile (*N* = 336)	33 (9.8)	78 (23.2)	8 (2.4)	65 (19.3)	152 (45.2)
Ecuador (*N* = 110)	19 (17.3)	37 (33.6)	3 (2.7)	25 (22.7)	26 (23.6)
Colombia (*N* = 254)	23 (9.1)	50 (19.7)	3 (1.2)	64 (25.2)	114 (44.9)
*P*-value	0.051	0.015	0.49	0.23	<0.001

The professionals who were diagnosed with COVID-19 reported changing rooms and self-isolating from loved ones more than those who did not get sick (*n* = 63 [36%] vs. *n* = 78 [20%], *p* < 0.001). Respondents were more likely to affirm that it was not necessary to take precautions at the residential level when they (*n* = 189, 48.5% versus *n* = 55, 31.4%, *p* < 0.001) or their family members did not get sick (*n* = 166, 47.8% versus *n* = 126, 35.7%, *p* = 0.001). The fact that a family member was infected had no effect on the frequency with which professionals considered alternative residential facilities necessary.

The professionals who worked in high-risk areas changed bedrooms to self-isolate more frequently than those working in low-risk areas (27.0% versus 19.8%, *p* = 0.026; Table 3).

Those who chose not to change their bedroom or residence expressed greater satisfaction with the support received from colleagues in their service/unit (7.9, 95% CI 7.6–8.1, *p* = 0.036) and in other services/units (6.6, 95% CI 6.3–6.9, *p* = 0.017). In addition, these respondents expressed greater satisfaction with the support received from their community (6.2, 95% CI 5.8–6.5, *p* = 0.015) and society at large (5.6, 95% CI 5.2–5.9, *p* < 0.001).

Chilean and Colombian professionals infected with SARS-CoV-2 perceived less need to change their room/residence than professionals infected in Ecuador (37.3 and 35.7% vs. 14.0%, *p* = 0.017).

The factors determining professionals’ decisions to self-isolate to protect the family were having an infected partner, being older, having been infected, working in a high-risk area, and having experienced a higher level of acute stress due to the pandemic (Table 4).

**Table 4:** Predictors of professionals’ decisions to self-isolate at home (*N* = 699).

	Exp(B)	95% CI		*P-*value
Having an infected partner	1.3	1.0	1.7	0.038
Older age	1.0	1.0	1.0	0.006
Infection with COVID-19	2.1	1.4	3.1	<0.001
Working in a high-risk area	1.4	1.0	2.1	0.059
Self-reported stress level (EASE scale)	1.1	1.0	1.1	<0.001

### 3.4. Impact of acute stress

Those who suffered a high level of acute stress tended to assign lower ratings to both the support perceived by the community (2.5, 95% CI 1.2–3.8) and the information on the pandemic situation received from their hospital/center (5.5, 95% CI 4.2–6.8) compared to those who reported low stress levels (community support: 5.0, 95% CI 4.8–5.2; *p* = 0.001; information: 6.6, 95% CI 6.4–6.8; *p* = 0.050). Professionals with high stress levels tended to seek alternative residential options to avoid infecting their relatives (Table 5), while a higher proportion of those who did not experience acute stress considered precautions at the residential level unnecessary (*n* = 290, 42.4% vs. *n* = 2, 12.5%, *p* = 0.019).

**Table 5:** Acute stress (results of EASE scale) and self-isolation measures during the early phases of the pandemic (*N* = 700).

	Yes Mean EASE score (95% CI)	No Mean EASE score (95% CI)	*P*-value
Changed residence	12.4 (10.9–13.9)	10.3 (9.8–10.8)	0.008
Changed bedrooms, self-isolating from loved ones	12.1 (11.1–13.0)	10.1 (9.5–10.6)	<0.001
Stayed in a residential facility for professionals	8.3 (5.2–11.4)	10.6 (10.1–11.1)	0.23
Considered but did not make residential changes	12.9 (11.9–13.8)	9.9 (9.3–10.4)	<0.001
Did not make or consider making any residential changes	8.1 (7.4–8.8)	12.3 (11.7–12.9)	<0.001

## 4. Discussion

This study aimed to determine, during the first wave of the COVID-19 pandemic, the satisfaction of healthcare professionals in Colombia, Chile, and Ecuador with the social support received by their colleagues and the community and the information provided by their institution, as well as their efforts to self-isolate as a preventive measure to avoid infecting loved ones, considering the effect of other variables such as sex, the experience of infection in first person or through a close colleague or relative, the risk associated with the activity and the healthcare area of destination, and the level of acute stress experienced. This measure complements the studies on the emotional impact experienced by professionals during this period (Rosales Vaca et al., 2022; Zhang et al., 2022) and provides clues for future crises about the value of social support to cope with highly demanding situations, such as those experienced during the first waves of the pandemic worldwide.

A quarter of the professionals surveyed were infected, and more than half had a relative or colleague who became ill with COVID-19. These figures could be related to the reported difficulties in obtaining personal protective equipment and the lack of adequate training during this phase of the pandemic (Martin-Delgado et al., 2020). Being infected with COVID-19 and working in a high-risk unit were associated with a worse assessment of the information and support received and a greater tendency to self-isolate to avoid infecting family members. This natural response to the uncertainty and fear of infection should be considered when preparing for future health emergencies to ensure that practitioners and trainees in the sector have adequate training to cope with such experiences.

Support among colleagues was valued positively, particularly by those not caring directly for COVID-19 patients. The positive effect of peer support in the healthcare community has been demonstrated in other critical situations, such as emergencies faced daily by first responders or the emotional and psychological impact of healthcare professional involvement in an adverse event or patient harm (Wade et al., 2022). Recognition of peers as a natural source of suitable support enables the structuring of peer support systems and resources that are economically viable and sustainable for healthcare institutions and strengthen the ability of teams to adaptively cope with highly stressful situations such as those experienced during the COVID-19 pandemic (Moran et al., 2020; Wu et al., 2020b). The poorest rating of the support received by professionals assigned to high-risk care areas (e.g., ICU, COVID-19) suggests some tensions among the most exposed personnel in the context of their work teams, a factor that must be considered in future health emergencies (Czyż-Szypenbejl et al., 2022). Middle managers’ styles and leadership are critical in these cases, as shown in other studies of teamwork problems during the pandemic (Obrien et al., 2021).

Regarding the perception of community support, the lower satisfaction expressed by professionals in the COVID-19 area and those infected with the virus could be explained by the social stigma associated with the risk of contagion (Taylor et al., 2020).

Geographical differences were also observed in the assessment of the support received from colleagues and society, with Chilean professionals reporting greater satisfaction. This variability by territory could be explained, in part, by the organizational climate and culture and, also, by the social values and beliefs of each country. Another factor to consider concerning this result is the epidemiological situation of each region at the time of the survey. In the specific case of Chile, the consultation of professionals coincided with the recovery phase after the fall in infections in the first wave, which may have had a positive effect on satisfaction, while, in Colombia, positive cases were booming (Martin-Delgado et al., 2022).

The data showing lower satisfaction with the support and information received among women versus men suggest that women may have received less information about the evolution of the pandemic, along with less support from colleagues, neighbors, and other social groups. This finding may respond to different expectations and behaviors in men and women (Huang et al., 2021). In both cases, the data point to gender-related determinants that affect the well-being of women (Liu et al., 2021), who had to face the same risks associated with patient care and returned to their homes just as men did, where they likely had to assume a greater number of domestic tasks than their male counterparts.

In general, healthcare professionals shared the perception that the information received about the pandemic from their institutions was poor. This result is not surprising, given that the management of COVID-19 at the global level demonstrated the lack of preparedness of all health systems for a crisis of this magnitude. The greater dissatisfaction of those who cared for COVID-19 patients and those who experienced the infection first-hand or through a close person may have been due to the greater need for information when facing situations of higher severity and uncertainty and suggests the non-existence or lack of dissemination of standardized protocols that would guide the correct action in each case (Koffman et al., 2020).

Feelings of acute stress were associated with a perceived need for more safety precautions when returning home and efforts to change bedrooms or residences to protect one’s family, constituting another precursor to self-isolation seeking. Of note, the infection of a family member with COVID-19 did not influence the adoption of protective measures at home, probably because the event to be avoided had already happened.

Those who did not feel the need to make changes in their residence were the same ones who expressed the highest satisfaction with the support received from colleagues and society. The information received about the evolution of the pandemic in the workplace did not influence their decision to self-isolate when they returned home. This finding is relevant, as it highlights the value of social support during a health emergency characterized by a high degree of uncertainty (Labrague, 2021). Moreover, the perceived need to self-isolate—an aspect that arose spontaneously in the early stages of the pandemic—should be considered when organizing future pandemic preparedness.

In summary, lack of support and information and the need to change residence were associated with exposure to higher-risk clinical settings (ICU and COVID-19 areas), SARS-CoV-2 infection, being female, and experiencing high levels of acute stress. These results are interesting in that they address the impact of the pandemic on healthcare professionals in three Latin American countries beyond the emotional and psychological consequences widely explored in previous studies. This work provides insight into less attended aspects that may be essential to enhance the response capacity of healthcare professionals and institutions in critical and highly demanding situations. This experience should serve for the development of institutional resources aimed at fostering professionals’ well-being, which can be structured under occupational health departments and incorporate peer support as one of the intervention strategies, as this is one of the preferred sources of support for the healthcare collective (Burlison et al., 2017; Wu et al., 2020a). In addition to this social support, an informational support strategy should be added. Information is the most potent tool against uncertainty and empowers healthcare professionals to make decisions even in complex situations (Wu et al., 2020b). At the individual level, interventions aimed at strengthening the resilience of healthcare professionals seem advisable. Most of these initiatives are based on mindfulness, physical activity, psychoeducation, social support, cognitive skills, emotional regulation, and relaxation (Pollock et al., 2020).

In short, the implications of this study for practice focus on the need to strengthen the resilience and response capacity of health systems and their human teams to prevent and mitigate the impact of possible future crises. To achieve this goal, involving and engaging healthcare managers and middle managers is crucial (Wu et al., 2020b; Obrien et al., 2021). Future studies should analyze the effect of these interventions on the resilience of health systems and professionals and their perception of the support and information received during future pandemics and other health crises.

### 4.1. Limitations

The sample was not random, so a self-selection bias among respondents who accepted the invitation to participate cannot be ruled out. In addition, convenience sampling was used, and participants were invited through institutional emails, instead of other methods that explicitly ensure randomization. The motivation and experience of professionals who decided whether to participate could differ, which would affect the generalizability of the results. In the analysis of the responses, it was not possible to control for the availability of resources, mental health support, the provision of personal protective equipment, and the incidence rates between and within countries or different health centers. These variables could affect respondents’ experiences. Nor was it possible to analyze the effect of the epidemiological situation in each country at the time of the survey on the professionals’ perception. The study covered 3 months, during which there were undoubtedly changes in the evolution of the pandemic and working conditions, which is likely to have introduced a bias in the results. It is also expectable that the epidemiological situation was different, not only between countries but also between territories and health areas within the same country. Thus, time and territory could have acted as confounding variables regarding the epidemiological situation when the healthcare professionals responded to the survey. Approximately a fifth of those surveyed did not know if they had been infected. When interpreting these data, it is important to recall the diagnostic limitations during the period analyzed (July–September 2020) and in the countries studied.

## 5. Conclusion

In the early stages of the COVID-19 pandemic, professionals in Chile, Colombia and Ecuador greatly valued the support received from their closest colleagues and peers, especially in the hospital setting. Being infected with COVID-19, working in high-risk areas, experiencing higher self-reported acute stress, and having an infected co-worker were predictors for deciding to voluntarily self-isolate at home to protect their relatives. The retrospective reading of what happened in the healthcare institutions of these three Latin American countries during the COVID-19 pandemic should serve to implement actions aimed at strengthening their response capacity and resilience and that of their staff and teams facing future crises. Peer support, staff well-being programs, institutional crisis communication strategies, the design and dissemination of protocols, and the commitment of managers and middle management are essential factors in addressing this challenge.

## Data availability statement

The raw data supporting the conclusions of this article will be made available by the authors, without undue reservation.

## Ethics statement

The studies involving humans were approved by the Pontific Catholic University of Chile Ethics Committee for Health Sciences (200630029). The studies were conducted in accordance with the local legislation and institutional requirements. Written informed consent for participation was not required from the participants or the participants’ legal guardians/next of kin because This study is based on a voluntary survey. By completing the survey, participants implicitly consent to take part. Contact information was provided so participants could withdraw from the study if they wished to do so.

## Author contributions

IC and JM designed and coordinated the research. RP, PS, and JM-D disseminated the study to collect data from participants. AG conducted the data codification and analyses. AG and JM interpreted the results. IC and JM wrote the draft of the manuscript. RP, PS, and JM-D reviewed and improved the manuscript. All authors read and approved the submitted version.

## Funding

This work was supported by the Conselleria de Sanitat Universal i Salut Pública (Generalitat Valenciana, Spain) and the European Regional Development Fund (ERDF) under the European Union Operational Program for the Valencian Community 2014–2020, within the framework of the REACT-EU action lines as the Union’s response to the COVID-19 pandemic [grant number FEDER-COVID-26]; and the Foundation for the Promotion of Health and Biomedical Research of Valencia Region (FISABIO) under the Grants for the development of research projects for consolidated research groups 2021 (grant number: UGP-21-215). During the development of this manuscript, JM enjoyed an intensified research activity contract from the Carlos III Health Institute (reference INT22/00012).

## Conflict of interest

The authors declare that the research was conducted in the absence of any commercial or financial relationships that could be construed as a potential conflict of interest.

## Publisher’s note

All claims expressed in this article are solely those of the authors and do not necessarily represent those of their affiliated organizations, or those of the publisher, the editors and the reviewers. Any product that may be evaluated in this article, or claim that may be made by its manufacturer, is not guaranteed or endorsed by the publisher.
